# Circulating FABP4 is eliminated by the kidney via glomerular filtration followed by megalin-mediated reabsorption

**DOI:** 10.1038/s41598-018-34902-w

**Published:** 2018-11-06

**Authors:** Suman Shrestha, Hiroaki Sunaga, Hirofumi Hanaoka, Aiko Yamaguchi, Shoji Kuwahara, Yogi Umbarawan, Kiyomi Nakajima, Tetsuo Machida, Masami Murakami, Akihiko Saito, Yoshito Tsushima, Masahiko Kurabayashi, Tatsuya Iso

**Affiliations:** 1 Department of Diagnostic Radiology and Nuclear Medicine, Gunma University Graduate School of Medicine, 3-39-22 Showa-machi, Maebashi, Gunma 371-8511 Japan; 2 Department of Cardiovascular Medicine, Gunma University Graduate School of Medicine, 3-39-22 Showa-machi, Maebashi, Gunma 371-8511 Japan; 3 Department of Bioimaging Information Analysis, Gunma University Graduate School of Medicine, 3-39-22 Showa-machi, Maebashi, Gunma 371-8511 Japan; 4 Department of Clinical Laboratory Medicine, Gunma University Graduate School of Medicine, 3-39-22 Showa-machi, Maebashi, Gunma 371-8511 Japan; 5 Research Program for Diagnostic and Molecular Imaging, Division of Integrated Oncology Research, Gunma University Initiative for Advanced Research (GIAR), 3-39-22 Showa-machi, Maebashi, Gunma 371-8511 Japan; 6 Program for obesity-related cardiovascular disease, Division of Endocrinology, Metabolism and Signaling, Gunma University Initiative for Advanced Research (GIAR), 3-39-22 Showa-machi, Maebashi, Gunma 371-8511 Japan; 7 Department of Applied Molecular Medicine, Niigata University Graduate School of Medical and Dental Sciences, 1-757 Asahimachi-dori, Chuo-ku, Niigata 951-8585 Japan; 8 Department of Internal Medicine, Faculty of Medicine Universitas Indonesia, Jl. Salemba Raya No. 6, Jakarta, 10430 Indonesia

## Abstract

Circulating fatty acid binding protein 4 (FABP4), secreted from adipocytes, is a potential biomarker for metabolic and cardiovascular diseases. Circulating FABP4 levels are positively associated with adiposity and adrenergic stimulation, but negatively with renal function. In this study, we addressed the issue of how the kidney regulates clearance of circulating FABP4. Tracing study revealed remarkable accumulation of ^125^I-labeled FABP4 in the kidney. Exogenous FABP4 was exclusively detected in the apical membrane of proximal tubule epithelial cells (PTECs). Bilateral nephrectomy resulted in marked elevation of circulating FABP4 levels. Accelerated lipolysis by β-3 adrenergic stimulation led to a marked elevation in circulating FABP4 in mice with severe renal dysfunction. Megalin, an endocytic receptor expressed in PTECs, plays a major role in reabsorption of proteins filtered through glomeruli. Quartz-crystal microbalance study revealed that FABP4 binds to megalin. In kidney-specific megalin knockout mice, a large amount of FABP4 was excreted in urine while circulating FABP4 levels were significantly reduced. Our data suggest that circulating FABP4 is processed by the kidney via the glomerular filtration followed by megalin-mediated reabsorption. Thus, it is likely that circulating FABP4 levels are determined mainly by balance between secretion rate of FABP4 from adipocytes and clearance rate of the kidney.

## Introduction

Fatty acid binding protein 4 (FABP4, also known as adipocyte FABP or aP2) is a member of FABP family (14–15 kDa proteins) and abundantly expressed in adipocytes and macrophages^1,2^. FABP4 is able to reversibly bind to hydrophobic ligands such as saturated and unsaturated long-chain fatty acids (FA), eicosanoids and other lipids, thus taking part in the regulation of lipid trafficking and responses at the cellular level. Clinical and animal-based studies have demonstrated that FABP4 functions as a critical mediator of inflammatory process both locally and systemically and has an important role in obesity-related metabolic diseases^1,2^.

FABP4 has also been introduced as a fat-derived circulating protein and a potent clinical biomarker for metabolic and cardiovascular diseases such as obesity, type 2 diabetes, hypertension, dyslipidemia, atherosclerosis, non-alcoholic fatty liver disease, ischemic heart disease, heart failure and renal failure in various cross-sectional and interventional studies^3,4^. It is likely that circulating FABP4 levels are regulated by three major factors: production in adipocytes, enhanced secretion via lipolysis and disposal probably via the kidney. Because majority of FABP4 is produced in adipocytes, circulating FABP4 levels are positively and strongly correlated with adiposity^4^. FABP4 is secreted from adipocytes despite the lack of an N-terminal secretory signal sequence via ER-Golgi-independent pathways^5–7^. Secretion of FABP4 is enhanced by lipolysis, which is accelerated during fasting and activation of sympathetic nervous system in a β-adrenergic receptors-dependent manner^5,6,8,9^. On the other hand, FABP4 is likely to be eliminated from the circulation mainly by renal clearance^10–12^. Circulating FABP4 levels are inversely associated with glomerular filtration rate (GFR) and markedly elevated up to 20-fold in patients undergoing maintenance hemodialysis. Compared to its production and enhanced secretion via lipolysis, however, little is known about precise mechanism by which FABP4 is removed from circulation via the kidney.

Kidney is a critical organ in clearance of many circulating proteins. When circulating macromolecules are <60 kDa, they are filtered through the glomeruli^13^. Larger proteins like albumin (66 kDa) and transferrin (81 kDa) are also filtered to a certain extent. It has been proposed that nearly all filtered plasma proteins are reabsorbed by the mechanism mediated by megalin and its associated molecule cubilin that are expressed in the apical membranes of proximal tubule epithelial cells (PTECs)^13,14^. Megalin is a giant, glycosylated protein (600 kDa) with similarities to endocytic receptors in the low-density lipoprotein (LDL) receptor family^15^. Megalin is heavily expressed in the brush border and endocytic vesicles for clearance of the filtrates in PTECs. Several animal studies support the notion that megalin serves as a multi-specific clearance receptor. Indeed, plenty of small proteins including circulating FABP1^16^, a member of FABP family secreted from liver, have been suggested to be reabsorbed by PTECs via megalin-dependent system^13,17–20^.

In this study, we demonstrate that a large amount of FABP4 filtered through glomeruli is reabsorbed by PTECs via megalin-mediated mechanism. We further showed that impairment of renal function in combination with accelerated lipolysis results in marked elevation of circulating FABP4 levels. Thus, we conclude that kidney is a key organ for clearance of circulating FABP4 via glomerular filtration of FABP4 and subsequent reabsorption by megalin-mediated endocytosis.

## Results

### Circulating FABP4 is quickly taken up by the kidney

**Figure 1:**
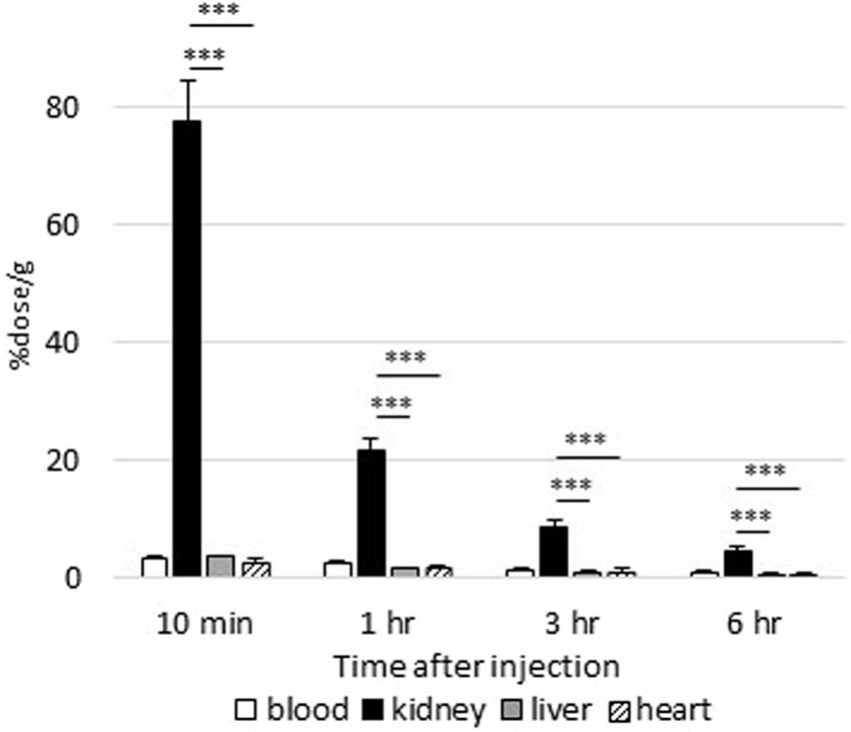
Circulating FABP4 is taken up by kidney. Blood, kidney, liver and heart were collected at indicated time points after intravenous injection of ^125^I-labeled recombinant FABP4 (^125^I-FABP4) to assess FABP4 uptake by each tissue. The data were expressed as the percent injected dose per gram tissue (%dose/g). n = 5. ***p < 0.001.

To determine which organ is responsible for clearance of circulating FABP4, we first studied accumulation of circulating FABP4 in several organs by intravenous injection (iv) of ^125^I-labeled recombinant FABP4 (^125^I-FABP4). Ten minutes after iv, ^125^I-FABP4 was markedly accumulated in the kidney compared to liver and heart and its levels declined thereafter (Figs 1 and S1). These findings strongly suggest that the kidney is the most crucial organ for clearance of circulating FABP4.

### Circulating FABP4 is accumulated in the apical membrane of PTECs

**Figure 2:**
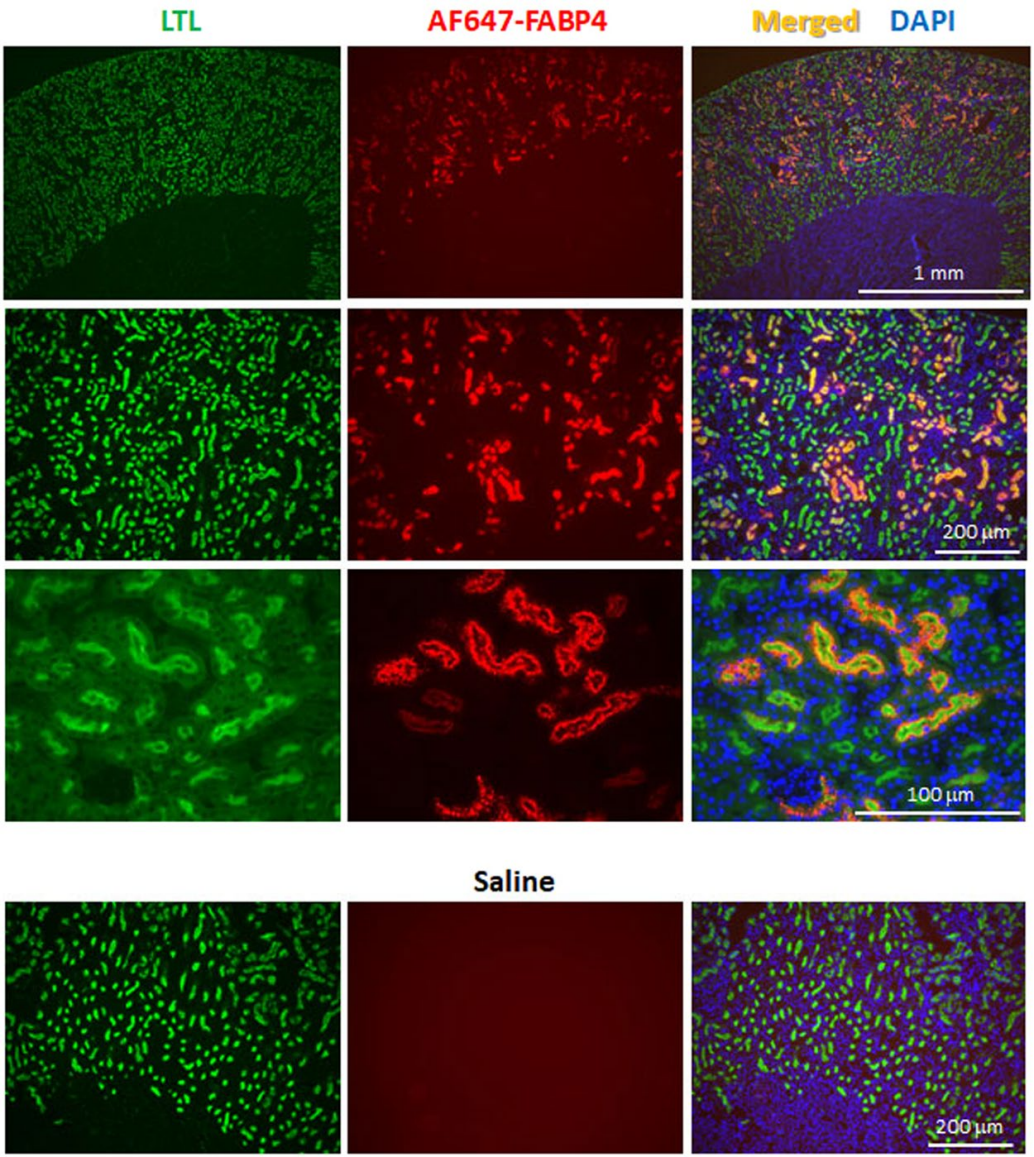
Circulating FABP4 is reabsorbed by PTECs. Kidney was isolated 10 min after intravenous injection of AF647-FABP4 (red) for subsequent immunofluorescence. Saline iv served as a negative control. LTL, Lotus Tetragonolobus Lectin, a marker for PTECs (green); DAPI, 4′,6-diamidino-2-phenylindole, a marker for nuclei (blue).

We next studied which nephron segment of kidney is involved in accumulation of circulating FABP4. Ten minutes after iv of AF647-labeled recombinant FABP4 (AF647-FABP4), kidney was isolated for subsequent immunofluorescence. AF647-FABP4 was exclusively accumulated in the apical membrane of PTECs that are detected by Lotus Tetragonolobus Lectin (LTL), a marker for PTECs (Fig. 2). AF647-FABP4-positive particles were also observed in cytoplasmic area below the apical membrane, suggesting endocytotic vesicles. Distribution of AF647-FABP4 was not overlapped with either Tamm-Horsfall Urinary Glycoprotein (THP), a marker for loop of Henle, or Calbindin-D (CalD), a marker for distal tubule epithelial cells (Fig. S2). These findings suggest that circulating FABP4 is filtered through the glomeruli and reabsorbed by PTECs after interaction with cell surface protein(s).

### Kidney has a significant role in clearance of circulating FABP4

**Figure 3:**
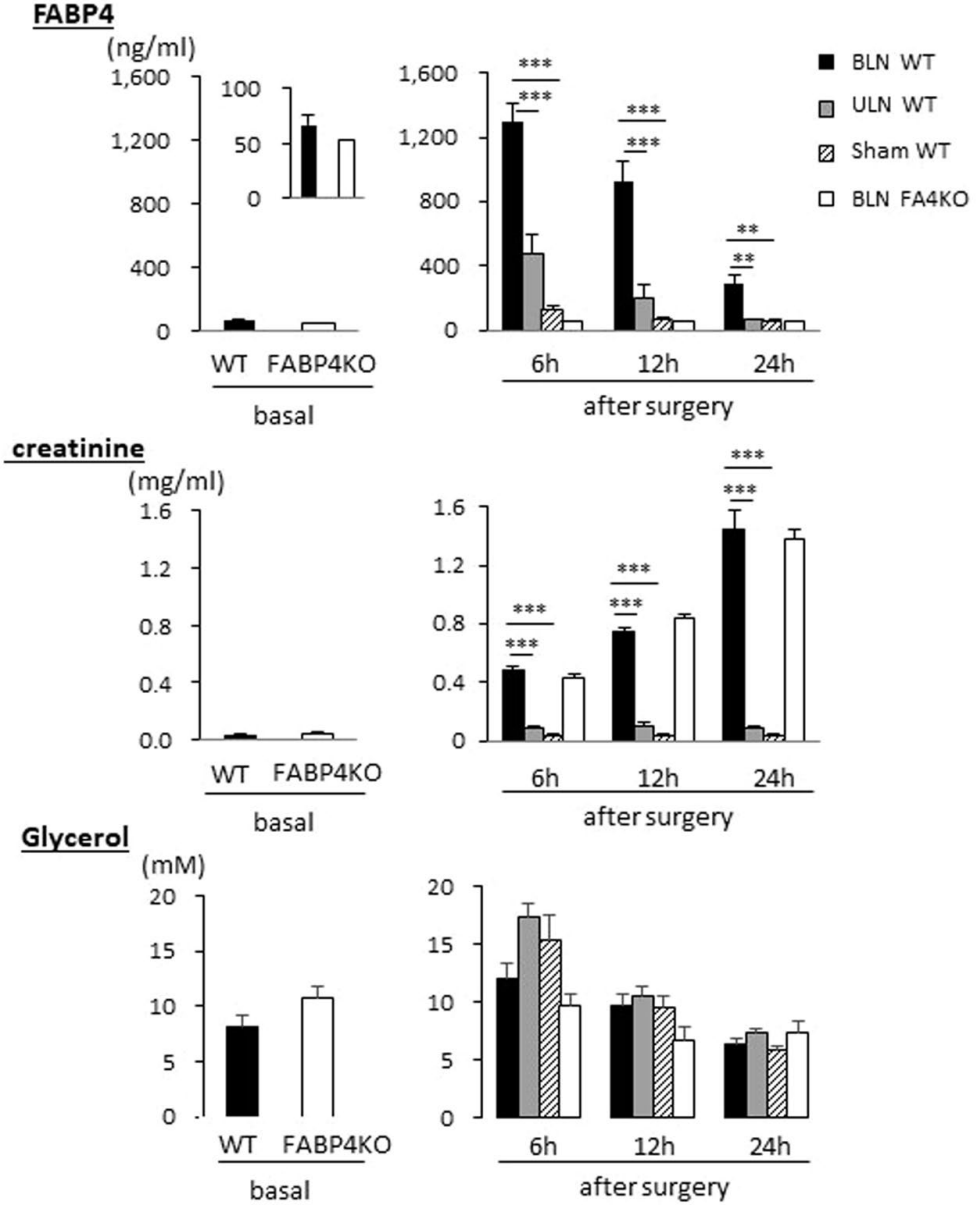
Circulating FABP4 levels were elevated by BLN. Blood was collected from retro-orbital plexus at indicated time points after surgery to measure serum levels of FABP4, creatinine and glycerol (right panels). FABP4 KO mice were used as negative control for serum levels of FABP4. Blood was also collected from non-treated mice as a control after a 6 h fast (left panels). n = 5. **p < 0.01 and ***p < 0.001. BLN, bilateral nephrectomy; ULN, unilateral nephrectomy.

**Figure 4:**
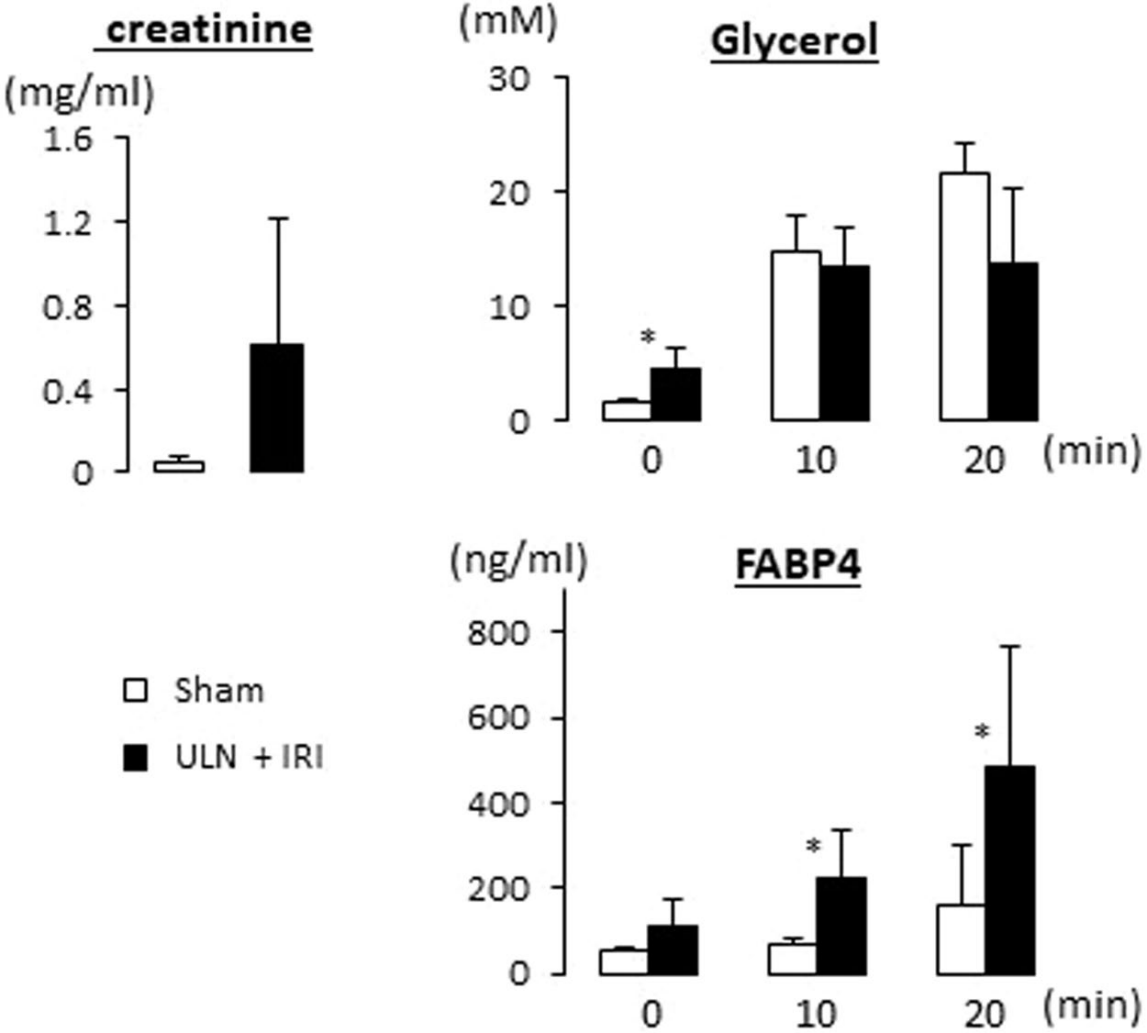
Circulating FABP4 levels were more elevated in mice model with severe renal dysfunction after intraperitoneal injection of CL316,243 (1 mg/kg). Blood was collected from retro-orbital plexus before, 10 and 20 minutes after injection to measure serum levels of creatinine, glycerol and FABP4. n = 5. * < 0.05. ULN, unilateral nephrectomy; IRI, ischemia reperfusion injury.

To study how a reduction of the glomerular filtration affects circulating FABP4 levels, bilateral and unilateral nephrectomy (BLN and ULN, respectively) were performed by using wild-type (WT) mice. Serum levels of FABP4 were markedly elevated 6 hours after BLN, and declined thereafter (Fig. 3). Serum levels of creatinine were gradually increased in BLN groups of both WT and FABP4 knockout (KO) mice (Fig. 3), which is consistent with abolishment of the glomerular filtration by BLN. Serum levels of glycerol were highest 6 hours after surgery in all groups and declined thereafter (Fig. 3), suggesting that damage of adipose tissue and/or activation of sympathetic nervous system peaked after surgery and decreased over time. These findings suggest that circulating FABP4 levels were markedly increased both by accelerated secretion of FABP4 into circulation and loss of the glomerular filtration. The data of ULN also provided useful information. Twenty-four hours after ULN, circulating FABP4 levels returned to the basal levels, suggesting that a single kidney is sufficient to maintain the basal FABP4 levels when secretion rate of FABP4 is low. In addition, serum levels of FABP4 in ULN group were prone to be higher compared to those in sham-operated group 6 and 12 hours after surgery. These findings suggest that secretion rate of FABP4 immediately after surgery exceeds FABP4 clearance rate of a single kidney. We further studied a combinational effect of renal dysfunction and acute enhancement of lipolysis by adrenergic stimulation on circulating FABP4 levels. To address the issue, we generated mice model with severe renal dysfunction by right ULN and ischemia reperfusion injury (IRI) on the left kidney. Serum creatinine levels were prone to be increased in mice with ULN plus IRI (Fig. 4). Elevation of serum levels of glycerol, a marker for lipolysis, by administration of CL316,243 (a β-3 adrenergic receptor agonist) was comparable between the two groups (Fig. 4). Serum levels of FABP4 were significantly elevated in mice with ULN plus IRI 10 and 20 min after administration of CL316,243 compared to those in sham-operated group (Fig. 4), suggesting that circulating FABP4 levels become more prominent when renal dysfunction and accelerated lipolysis are combined. Taken together, our data strongly suggest that serum levels of FABP4 are determined by balance between secretion rate of FABP4 from adipocytes and clearance rate of the kidney.

### Megalin is involved in reabsorption of FABP4 filtered through the glomeruli

**Figure 5:**
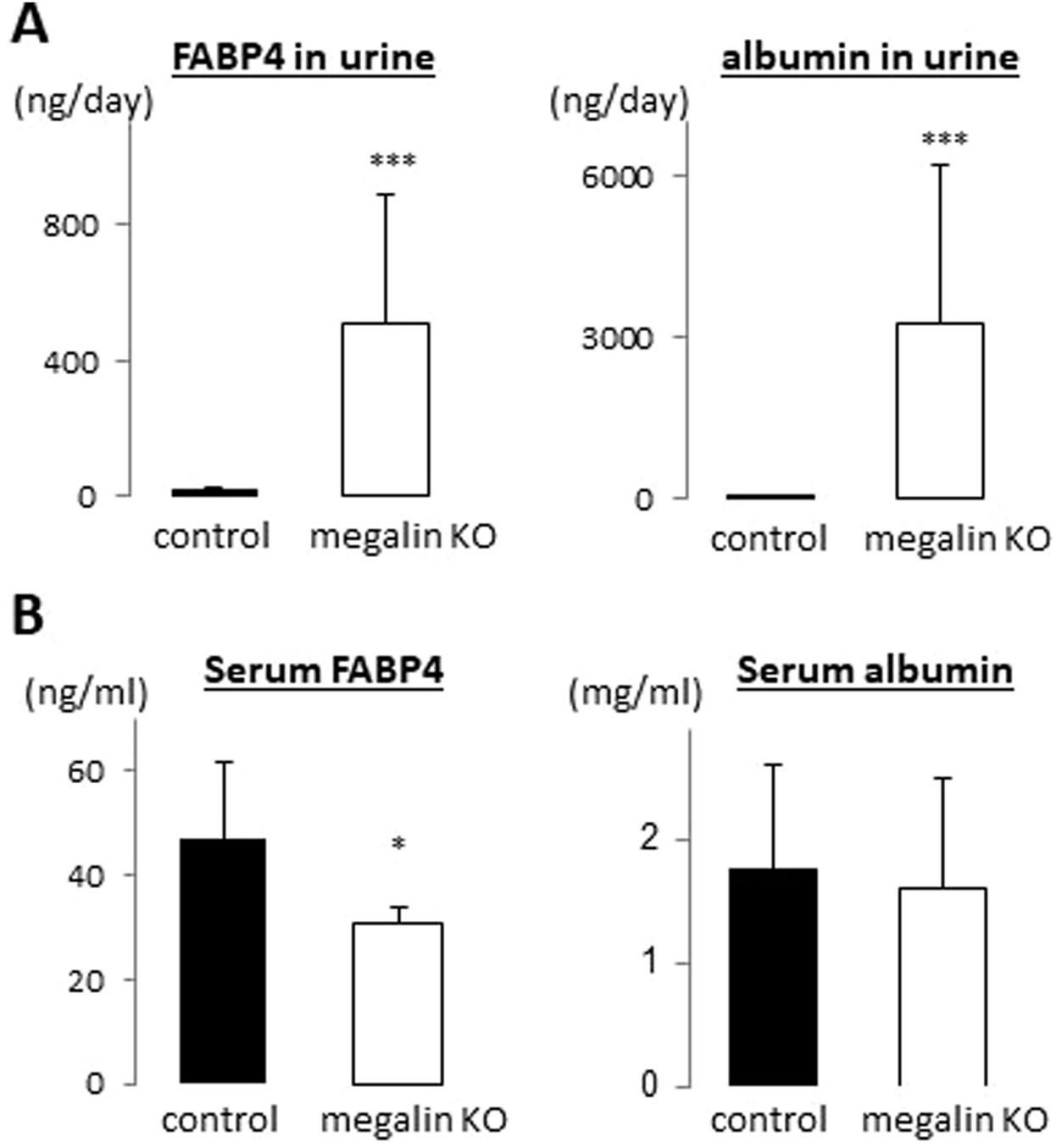
FABP4 is excreted to urine in kidney-specific megalin-KO mice (NDRG1 *cre* ERT2, megalin *flox/flox*). (**A**) Levels of FABP4 and albumin in urine. Note that FABP4 levels were markedly elevated in urine in megalin KO mice while it was hardly detected in urine in control (megalin *flox/flox*). (**B**) Levels of FABP4 and albumin in serum. n = 5. *p < 0.05 and ***p < 0.001.

**Figure 6:**
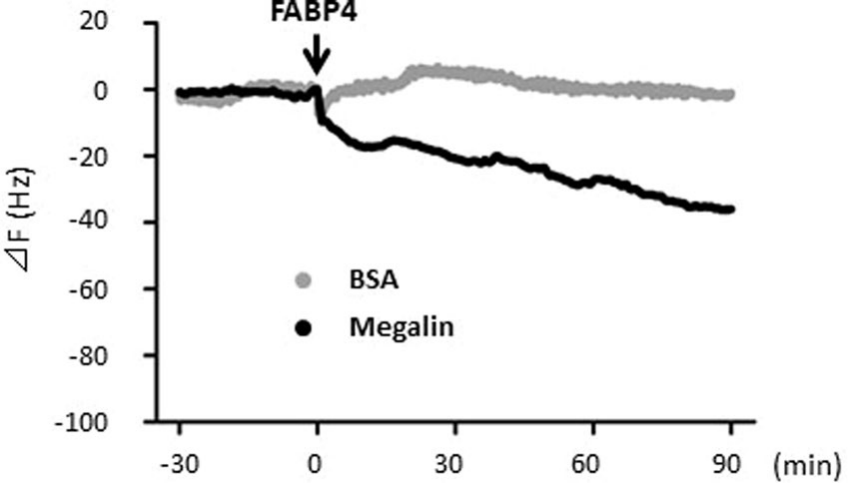
QCM analysis for the binding of FABP4 to megalin. FABP4 (1 mg/ml) was injected at the time point indicated by the arrow into the QCM chamber in which megalin or FA-free BSA had been immobilized on the sensor chips. The binding of FABP4 to the chip-immobilized protein is indicated by a change in the frequency, ΔF. All the experiments were repeated at least three times and the representative result is shown. Note that FABP4 bound to megalin, but not to albumin.

We next studied what molecule is involved in FABP4 clearance in the kidney. Oyama *et al*. have reported the possibility that FABP1, a member of FABP family and secreted mainly from liver into circulation, is taken up by the kidney through a giant plasma membrane protein megalin^16^, which plays a major role in reabsorption of the filtrates. Consistent with megalin distribution in the kidney, immunofluorescence revealed that exogenous FABP4 was accumulated in the PTECs in the kidney (Fig. 2). To study whether megalin is involved in reabsorption of FABP4 by the kidney, levels of FABP4 in urine and serum were measured by using kidney-specific megalin KO mice (hereafter megalin KO). FABP4 levels were markedly elevated in urine in megalin KO mice while it was hardly detected in control mice (Fig. 5A). A large amount of albumin in urine was also detected as reported previously^17^. Consistent with excretion of FABP4 into urine, serum levels of FABP4 in megalin KO mice were reduced compared to those in control (Fig. 5B). *In vitro* protein-protein interaction between FABP4 and megalin was further confirmed by QCM (Fig. 6). These findings suggest that megalin expressed on the apical membrane of PTECs is a responsible molecule to reabsorb FABP4 after the glomerular filtration.

## Discussion

Levels of circulating proteins are regulated by production, secretion and clearance in general. We showed in this study that majority of circulating FABP4, produced and secreted from adipocytes, is eliminated by the kidney. Exogenous FABP4 was exclusively accumulated in the apical membrane of PTCEs where magailn is expressed. It is confirmed that FABP4 directly binds to megalin *in vitro*. In kidney-specific megalin KO mice, reabsorption of FABP4 was disturbed, resulting in the excretion of a large amount of FABP4 in urine with a reduction in serum levels of FABP4. BLN and ULN revealed that clearance rate of FABP4 by the kidney is a crucial factor for serum levels of FABP4, which is also greatly influenced by secretion rate of FABP4 by surgical damage of adipocytes and accelerated lipolysis by β-3 adrenergic stimulation. Our data suggest that clearance of circulating FABP4 depends on the glomerular filtration of FABP4 and subsequent reabsorption mediated by megalin in PTECs. We further suggest that serum levels of FABP4 are determined mainly by balance between secretion rate of FABP4 from adipocytes and clearance rate of the kidney.

### Close association between reduced GFR and elevation of circulating FABP4

The glomerular sieving of proteins is closely linked with their reabsorption via PTECs. Nearly all the filtrated proteins are reabsorbed, resulting in little proteinuria in the normal kidney^13^. Renal filtration combined with reabsorption is also an important route of elimination of FABP4. In kidney-specific megalin KO mice, reabsorption of FABP4 is lost with no change in the glomerular filtration, which causes a reduction in circulating FABP4. On the other hand, elevation of circulating FABP4 occurs when GFR is markedly reduced or abolished. Circulating FABP4 is negatively correlated with GFR and markedly increased up to 20-fold in patients undergoing maintenance hemodialysis^8,10–12^. We showed that BLN induced marked elevation of circulating FABP4. Thus, loss of reabsorption alone causes a decrease in serum FABP4 levels while a reduction in GFR plays a major role in elevation of circulating FABP4. These findings strongly support the notion that the kidney is a major organ for disposal of circulating FABP4.

### Ligand-specificity by megalin/cubilin-mediated endocytosis

Megalin mediates the endocytosis of a highly diverse group of ligands including peptides, enzymes, hormones and plasma proteins such as vitamin-binding proteins and hormone-binding proteins^13,14^. Some of the ligands are shared with cubilin, whereas others are specific for either megalin or cubilin. To date, it has become clear that efficient reabsorption of the filtered proteins is mediated by these two membrane receptors. Little proteinuria occurs in normal subjects, suggesting that all proteins filtered through glomeruli is reabsorbed by PTECs without any ligand-specificity. Therefore, it is possible that megalin/cubilin mediated endocytosis contributes to reabsorption of most small filtered proteins (<60 kDa). In this aspect, molecular size of FABP family (14–15 kDa) suggests that all FABP members are potential targets for megalin/cublin-mediated endocytosis. Indeed, we showed in this study that circulating FABP4 is reabsorbed by the kidney in a megalin-dependent manner. In addition, serum levels of other FABP members such as FABP1 to 5 have been shown to be inversely correlated with GFR^21^, suggesting kidney-dependent clearance. Moreover, it is also reported that FABP1 is a possible target for megalin-mediated clearance^16^. Together, these findings suggest that megalin/cubilin-mediated reabsorption by PTECs is a common pathway with less ligand-specificity for disposal of circulating small molecules including FABP family members.

### Fate of circulating proteins after the endocytosis by PTECs

It has been generally considered that ligands reabsorbed by megalin/cubilin complex in the PTECs are degraded in the lysosome and then the degraded products are secreted into circulation via basolateral transport^13,14^. However, there is controversy whether filtered and reabsorbed proteins, especially albumin, are exclusively subjected to lysosomal degradation into amino acids or whether a fraction of intact proteins is returned to the circulation. Recently, Tenten *et al*. reported that reabsorbed albumin is protected from lysosomal degradation and recycled intact^22^. Nephrotic syndrome excreting a large amount of albumin in urine causes hypoalbuminemia, which also suggests that albumin recycling following reabsorption plays a role to maintain serum levels of albumin. In this study, we showed that circulating FABP4 levels were decreased in megalin KO mice, which was induced by abundant excretion of FABP4 in urine. These findings suggest a possible scenario that a large amount of circulating FABP4 is filtered through glomerulus and that a fraction of reabsorbed FABP4 is recycled intact or serves as materials for de novo FABP4 synthesis (Fig. S3). Our data showed at least that mice cannot maintain basal levels of circulating FABP4 without megalin-mediated reabsorption. Further studies are needed to investigate whether protein recycling without degradation in lysosome is applied to only limited proteins or a large number of proteins including FABPs.

### Disposal of circulating proteins by other routes

The albumin turnover time reflects a liver synthesis rate balanced by the albumin clearance rate^23^. In addition to renal clearance (<6% of albumin synthesis), circulating albumin is removed by gastrointestinal and catabolic clearance (10% and 84% of albumin synthesis, respectively) in normal humans^23^. Although specifics of catabolic clearance remain to be unsolved, approximately 60% of the albumin catabolism occurs in skin and muscle, primarily in the fibroblasts of these organs^23^. Thus, renal clearance rate of albumin is very small compared to gastrointestinal and catabolic clearance, which can account for no change in serum levels of albumin in megalin-KO mice (Fig. 5B, right panel). Compared to albumin in serum, circulating FABP4 levels were significantly reduced in megalin KO mice (Fig. 5B, left panel). In addition, circulating FABP4 levels were markedly elevated 24 hours after BLN. These findings suggest a predominant role of the kidney in FABP4 clearance. However, elevation of FABP4 by BLN declined over time and there is upper limit of circulating FABP4 levels in patients undergoing maintenance hemodialysis^10,12^. These findings suggest that some routes other than the kidney may exist for FABP4 clearance. It is needed to study what other mechanisms are involved in FABP4 clearance in the future.

## Materials and Methods

All procedures involving animals were approved by the Institutional Animal Care and Use Committee in Gunma University and Niigata University. All experiments were performed in accordance with the NIH guidelines (Guide for the Care and Use of Laboratory Animals).

### Mice

For tracing studies with isotope-labeled and fluorescence-labeled FABP4, male ddY mice were purchased from Japan SLC, Inc. (Hamamatsu, Japan) for usability for intravenous injection of tracers via tail vein. Male kidney-specific megalin KO mice (NDRG1 *cre* ERT2, megalin *flox*/*flox*)^17^ maintained on the C57BL/6J background as well as their littermate male control mice (megalin *flox/flox*) were used to study excretion of FABP4 in urine. To delete the megalin gene in adulthood, 175 mg/kg body weight of tamoxifen (T5648, Sigma-Aldrich, MO) dissolved in sunflower oil/ethanol (11:1) (17.5 mg/mL) was gavaged to mice for 5 days a week at their age of 7 and 9 week-old. For nephrectomy experiments, WT C57BL6J male mice were purchased from Japan SLC, Inc. (Hamamatsu, Japan). Mice deficient for *Fabp4* (FABP4 KO) with the C57BL6J background served as negative control^24^. The ages (10 to 14 weeks) and body weights (22 to 32 grams) of mice were matched for all experiments. The mice were housed in a temperature-controlled room in a 12-h light/12-h dark cycle and had unrestricted access to water and standard chow (CE-2, Clea Japan, Inc.). Urine was collected for 24 hours in metabolic balance cages and stored at −20 °C until use. Blood was collected from retro-orbital plexus to measure biochemical parameters and centrifuged at 1,500 × *g* for 15 minutes at 4 °C to separate the serum, which was stored at −20 °C until use.

### Biodistribution of ^125^I-FABP4

Recombinant human FABP4 (ab133145, abcam, MA) was labeled with Na^125^I (NEZ033A, Perkin Elmer, MA) by Chloramine T method^25^. One hundred µL of ^125^I-FABP4 (4 kBq) was injected into the ddY mice intravenously via the lateral tail vein and the mice were euthanized 10 minutes, 1, 3 and 6 hours after injection. After blood sampling, kidney, heart and liver were isolated, weighed and subjected to well-type gamma counter (ARC-7001; Hitachi Aloka Medical, Tokyo) for measurement of radioactivity.

### Immunofluorescence analysis

Recombinant human FABP4 was labeled with Alexa Fluor 647 reactive dye (AF647, A37573, Thermo Fisher Scientific, MA) according to the manufacturer’s protocol^26^. Ten minutes after intravenous injection of AF647-FABP4 (15 μg/mouse in 200 μl of saline) or saline alone, kidneys were isolated, longitudinally cut into two equal halves, fixed in Carnoy’s solution (60% ethanol, 30% chloroform and 10% glacial acetic acid) for 6 hours and embedded in paraffin. Immunofluorescence analysis was performed with fluorescein LTL (FL-1321, Vector laboratories, CA) or primary antibodies against THP (sc-19554, Santa Cruz, TX) or CalD (ab82812, abcam, MA). Anti-goat and anti-mouse secondary antibodies conjugated with Alexa Fluor 488 (A-110055 and R37120, respectively, Thermo Fisher Scientific, MA) were used to detect THP and CalD, respectively. Images for immunofluorescent analysis were captured with Biozero-8100 immunofluorescence microscope (Keyence Corporation, Osaka, Japan).

### Nephrectomy and blood sampling

BLN, ULN and sham operation were performed as described previously^27^. In brief, mice were anesthetized with intraperitoneal injection of a combination of ketamine hydrochloride and xylazine hydrochloride. The kidneys were exposed from the flank, and removed after ligation of the pedicles. The same surgical treatments except kidney removal and pedicle ligation were carried out for both kidneys of sham group and the other side of the removed kidney of ULN group. Blood was collected from retro-orbital venous plexus 6, 12 and 24 hours after surgery.

### ULN, ischemia reperfusion injury (IRI) and administration of CL316,243

To generate mice model with severe renal dysfunction, C57BL6J male mice were subjected to right ULN and subsequent left IRI^28^. In brief, on the next day after right ULN, the left kidney was exposed from the flank and then the pedicle was clamped using a non-traumatic vascular clamp for 30 minutes. Ischemia and reperfusion of the kidneys were confirmed visually. On the next day after IRI surgery, blood was collected from retro-orbital venous plexus before, 10 and 20 minutes after intraperitoneal injection of CL316,243 (C5976, Sigma-Aldrich, MO), a β3-adrenergic receptor agonist.

### Measurement of serum FABP4 and biochemical parameters

Serum FABP4 levels were measured with mouse FABP4 enzyme-linked immunosorbent assay (ELISA) kit according to the manufacturer’s protocol (RD291036200R, BioVendor Research and Diagnostic Products, Czech Republic)^9,23^. Blood glucose was measured by glutest sensor (Sanwa Kagaku, Aichi, Japan). Serum levels of glycerol (Colorimetric assay kit, K630-100, Biovision, CA) were measured according to the manufacturer’s protocol. Serum levels of creatinine were measured using LABOSPECT 008 analyzers (Hitachi High-Technologies Corporation, Tokyo).

### Quartz-crystal microbalance (QCM)

The binding of FABP4 to megalin was examined using a highly sensitive 27 MHz QCM (Affinix Q8) (ULVAC, Kanagawa, Japan) as described previously^16^. In brief, the rat megalin, which had been prepared from renal microvillar membranes by immunoaffinity chromatography, at a concentration of 10 mg/mL in buffer B (10 mM HEPES, pH 7.4, 150 mM NaCl, 2 mM CaCl_2_), or FA-free BSA (Sigma-Aldrich, MO) in the negative control experiment, was immobilized on the sensor chips. FABP4 (1 mg/mL) was injected into the chamber for binding to megalin or albumin. The resonance frequency of the QCM was defined as the 0 position after equilibrium. The stability and drift of the 27 MHz QCM frequency in the solution were 73 Hz. The binding affinity was determined from the frequency changes upon cumulative injection of a small volume (200 μL) of 1 mg/mL FABP4.

### Statistical analysis

Statistical analysis was carried out in SPSS version 24.0 (SPSS, Chicago, IL, USA) and the numeric data are presented as mean ± standard deviation (SD). The statistical analysis was performed using an unpaired Student’s t-test for 2 groups. A one-way ANOVA with Bonferroni’s post hoc multiple comparison tests were performed for the 3 groups analysis. A p value < 0.05 was considered to be statistically significant. *p < 0.05, **p < 0.01 and ***p < 0.001.

## Electronic supplementary material

Supplementary Information

## Data Availability

The datasets used and/or analyzed during the current study available from the corresponding author on reasonable request.
