# Supplementary material for: Circulating FABP4 is eliminated by the kidney via glomerular filtration followed by megalin-mediated reabsorption
Source: Sci Rep. 2018 Nov 6;8:16451. doi: 10.1038/s41598-018-34902-w (PMC6219568; doi:10.1038/s41598-018-34902-w)
Supplement: Supplementary file 1 — Supplementary Information [file 41598_2018_34902_MOESM1_ESM.pdf]

Supplementary Information

Circulating FABP4 is eliminated by the kidney via glomerular filtration followed by megalin-mediated reabsorption

Suman Shrestha, Hiroaki Sunaga, Hirofumi Hanaoka, Aiko Yamaguchi, Shoji Kuwahara, Yogi Umbarawan, Kiyomi Nakajima, Tetsuo Machida, Masami Murakami, Akihiko Saito, Yoshito Tsushima, Masahiko Kurabayashi, Tatsuya Iso

Supplementary materials and methods

Immunofluorescence analysis

The method for immunofluorescence analysis is described in the main text.

Supplementary figure legend

Figure S1

Circulating FABP4 is reabsorbed by PTECs. Kidney was isolated 10 min after intravenous injection of AF647-FABP4 (red) for subsequent immunofluorescence. Enlarged photographs are shown to confirm FABP4-positive particles below LTT-positive plasma membrane. LTL, Lotus Tetragonolobus Lectin, a marker for PTECs (green); DAPI, 4',6-diamidino-2-phenylindole, a marker for nuclei (blue).

Figure S2

Kidney was isolated 10 min after intravenous injection of AF647-FABP4 (red) for subsequent immunofluorescence. THP, Tamm-Horsfall Urinary Glycoprotein, a marker for loop of Henle (green); CalD, Calbindin-D, a marker for distal tubules (green); DAPI, 4',6-diamidino-2-phenylindole, a marker for nuclei (blue).

Figure S3

Degradation and recycling of FABP4 after megalin-mediated reabsorption. (A) In WT mice, FABP4 filtered through glomeruli is nearly 100% reabsorbed via megalin-mediated mechanism, resulting in no FABP4 in urine. After reabsorption, majority of FABP4 is degraded while a certain amount of FABP4 (X) is recycled into blood. It is also possible that megalin-independent reabsorption of FABP4 (α) is present. This scenario is supported by findings observed in tracer study with

^{125}I-FABP4 and a reduction in serum FABP4 in megalin KO mice. (B) A large amount of FABP4 was excreted in urine in megalin KO mice, indicative of defective FABP4 reabsorption via megalin. Marked accumulation of ^{125}I-FABP4 in kidney and subsequent reduction in kidney and blood over time in WT mice (figure 1) strongly suggest that circulating FABP4 is degraded in kidney. On the other hand, a reduction in serum FABP4 in megalin KO mice (figure 5A) suggests that recycling of FABP4 after reabsorption is necessary to maintain serum levels of FABP4. If there was no recycling in WT, a reduction in serum FABP4 could not occur in megalin KO mice. Note that amount of FABP4 reabsorbed via megalin was set at 100 and that megalin-independent reabsorption of FABP4 (α) may be also present.

Figure S1

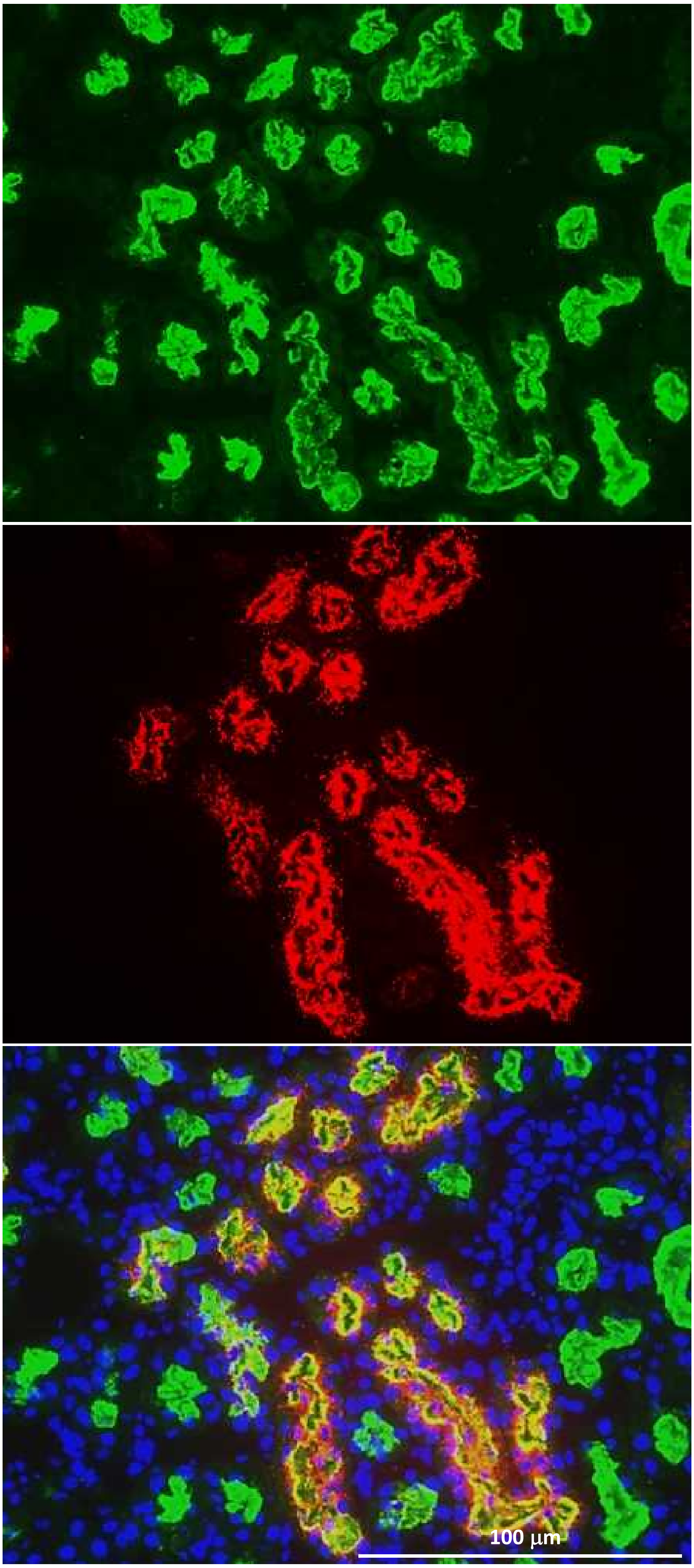

LTL

AF647-FABP4

LTL
AF647-FABP4
Merged
DAPI

Figure S2

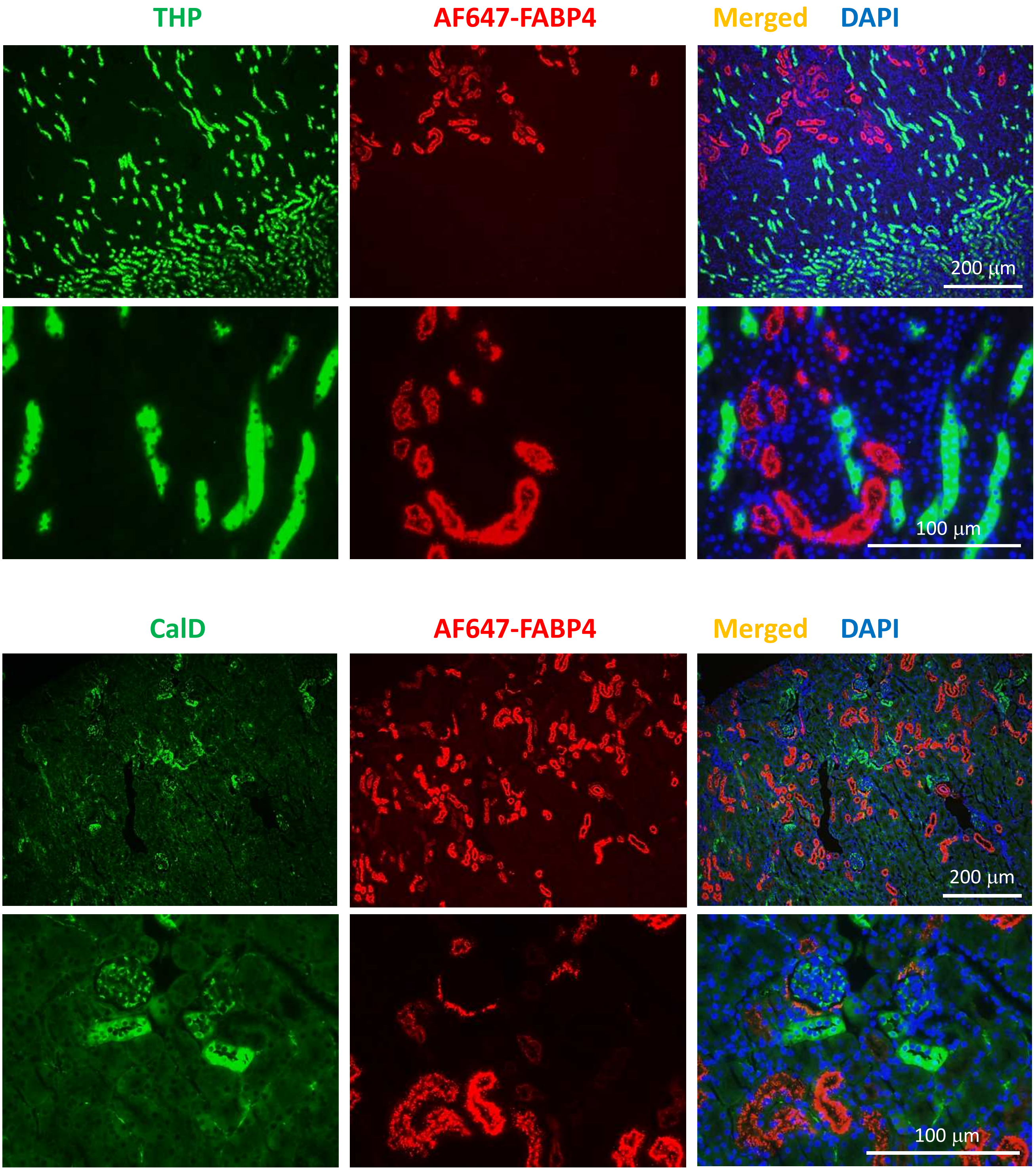
THP
AF647-FABP4
Merged
DAPI
200 µm
100 µm
CalD
AF647-FABP4
Merged
DAPI
200 µm
100 µm

Figure S3

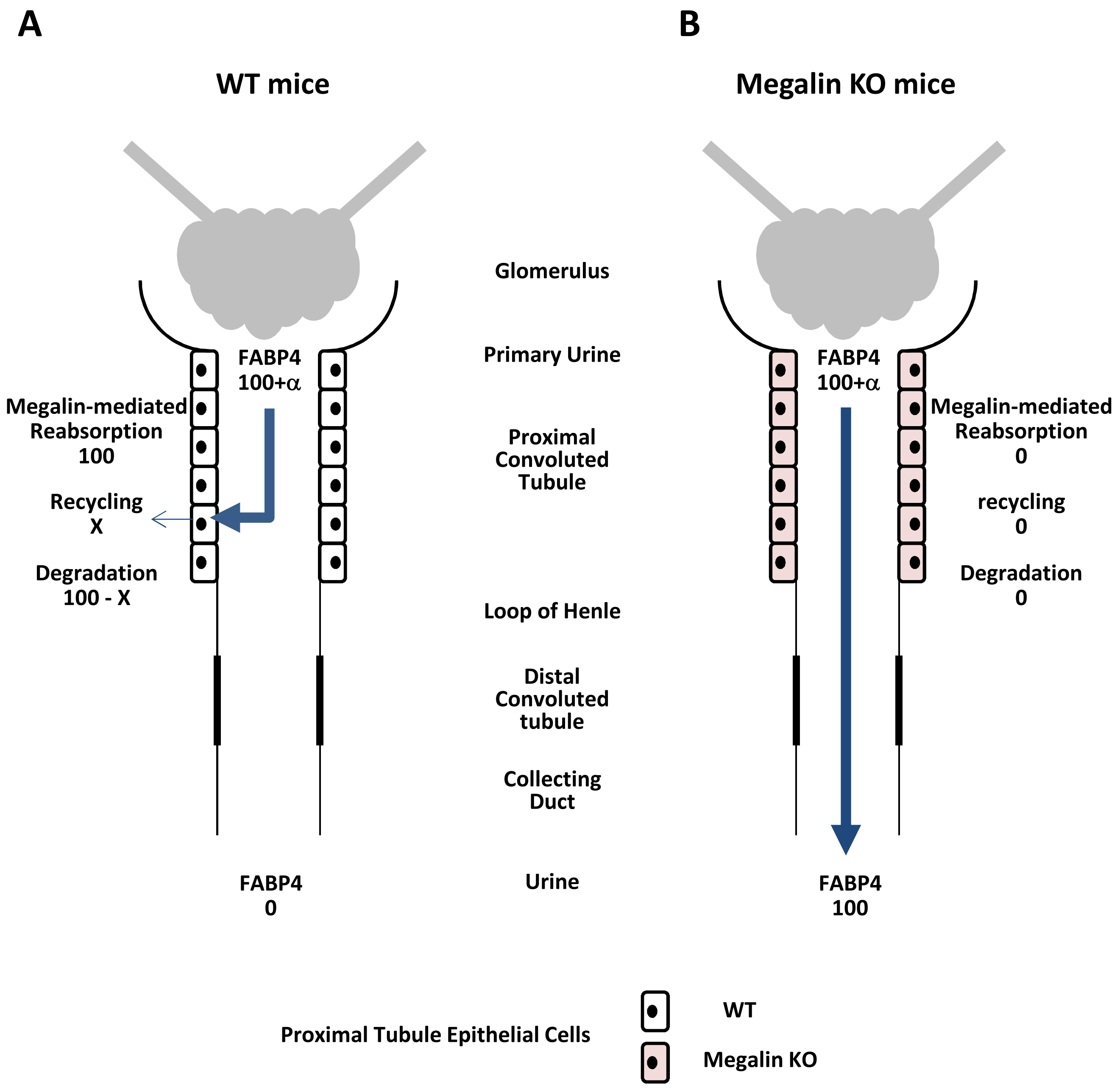

A
WT mice
B
Megalin KO mice
Glomerulus
Primary Urine
FABP4
100+α
Megalin-mediated
Reabsorption
100
Recycling
X
Degradation
100 - X
Proximal
Convoluted
Tubule
FABP4
100+α
Megalin-mediated
Reabsorption
0
recycling
0
Degradation
0
Loop of Henle
Distal
Convoluted
tubule
Collecting
Duct
FABP4
0
Urine
FABP4
100
Proximal Tubule Epithelial Cells
WT
Megalin KO
